# Effect of physical exercise on markers of neuronal dysfunction in cerebrospinal fluid in patients with Alzheimer's disease

**DOI:** 10.1016/j.trci.2017.03.007

**Published:** 2017-04-17

**Authors:** Camilla Steen Jensen, Erik Portelius, Peter Høgh, Lene Wermuth, Kaj Blennow, Henrik Zetterberg, Steen Gregers Hasselbalch, Anja Hviid Simonsen

**Affiliations:** a Danish Dementia Research Centre, Department of Neurology, Rigshospitalet University of Copenhagen, Copenhagen, Denmark; b Department of Psychiatry and Neurochemistry, Institute of Neuroscience and Physiology, The Sahlgrenska Academy at the University of Gothenburg, Mölndal, Sweden; c Clinical Neurochemistry Laboratory, Sahlgrenska University Hospital, Mölndal, Sweden; d Regional Dementia Research Centre, Department of Neurology, Zealand University Hospital and Copenhagen University Hospital, Roskilde, Denmark; e Dementia Clinic, Department of Neurology, Odense University Hospital, Odense, Denmark; f Department of Molecular Neuroscience, UCL Institute of Neurology, London, UK

**Keywords:** Alzheimer's disease, Physical exercise, Biomarkers, Synapses, Neurons, Stability

## Abstract

**Introduction:**

Physical exercise has gained increasing focus as a potential mean to maintain cognitive function in patients with Alzheimer's disease (AD). Alongside the markers of specific AD pathology (amyloid β and tau), other pathologies such as neuronal damage and synaptic loss have been proposed as markers of the disease. Here, we study the effect of physical exercise on biomarkers of neuronal and synaptic integrity.

**Methods:**

Cerebrospinal fluid (CSF) from 51 AD subjects who participated in the randomized controlled trial Preserving Cognition, Quality of Life, Physical Health and Functional Ability in Alzheimer's Disease: The Effect of Physical Exercise (ADEX) was analyzed for the concentration of neurofilament light (NFL), neurogranin (Ng), visinin-like protein-1 (VILIP-1), and chitinase-3–like protein 1 (YKL-40). Participants were subjected to either 16 weeks of moderate- to high-intensity exercise (*n* = 25) or treatment as usual (control group, *n* = 26), and CSF was collected before and after intervention.

**Results:**

No significant differences in CSF concentrations of VILIP-1, YKL-40, NFL, and Ng were observed when comparing mean change from baseline between the exercise and control groups. Similarly, when classifying subjects based on their exercise levels, no significant changes were observed for the biomarkers in the control group compared with the high-exercise group (attending 80% of the exercise sessions with an intensity of 70% of maximum heart rate or above).

**Discussion:**

These results are not supportive of a modulatory effect of physical exercise on the selected biomarkers of neuronal and synaptic integrity in patients with AD.

## 1 Introduction

The molecular pathological mechanisms behind Alzheimer's disease (AD) have been studied for decades. Although the exact mechanisms are still not quite clear, accumulation of amyloid β 42 (Aβ42) is an early pathological hallmark that interacts with and/or promotes neurodegeneration without being directly linked to clinical symptoms [1]. One key feature of neurodegeneration in AD is neuronal and synaptic loss that better reflects AD progression and cognitive decline [1], [2], [3]. Studies in transgenic animals suggest effects of physical exercise on neurogenesis, cognition, and amyloid deposition [4]. Further, epidemiological studies suggest that higher physical activity may reduce the risk of dementia in late life [5], and some intervention studies in elderly suggest that participation in physical activity and cognitive training may reduce future cognitive decline [6]. However, these studies have investigated possible effects on risk of dementia and cognitive decline in general and have not used any biomarkers to diagnose AD or specifically study effects on AD pathophysiology. Furthermore, in an effort to arrest the decline in cognitive function (CF) and loss of activities of daily living, nonpharmacological approaches have been investigated. In the randomized clinical trial “Preserving Cognition, Quality of Life, Physical Health and Functional Ability in Alzheimer's Disease: The Effect of Physical Exercise (ADEX) study,” we analyzed the effect of 16 weeks moderate- to high-intensity physical exercise (EXE) in patients with AD on a cognitive [7], functional [8], and molecular level [9]. Despite some effect on clinical symptoms such as processing speed and neuropsychiatric symptoms [7], we found no effect of EXE on levels of Aβ, tau, and phosphorylated tau (p-tau) in cerebrospinal fluid (CSF) [9]. Therefore, we sought to investigate downstream molecular processes that potentially could be influenced by EXE. In this study, we investigated the effect of EXE on molecular markers of AD neurodegeneration in CSF from patients with AD participating in the ADEX study. This is not a study on the potential diagnostic value of these markers as in cross-sectional study, however an explorative study on modulating effects of selected markers on exercise. Neurofilament light (NFL) is a marker of damage to large-caliber myelinated axons, and elevated CSF levels have been associated with cognitive deterioration and structural changes in the white matter and brain atrophy in patients with AD [10]. Elevated CSF NFL is not a specific AD marker, and CSF NFL levels do not correlate with Aβ42 levels, indicating that the changes in NFL are not driven by Aβ42 pathology [10]. Similar to NFL, CSF neurogranin (Ng) levels do not correlate with Aβ42 levels in patients with mild cognitive impairment (MCI) and AD [11], [12]. The synaptic protein Ng is released to the CSF from the synapses, which is an early event in the pathogenesis of AD. This also makes Ng a candidate marker for early AD diagnosis. Alongside this, Ng possesses the ability to differentiate patients with MCI who progress to AD (elevated Ng levels) and patients with MCI who remain stable (low levels of Ng) [11], [12]. Just as Ng, the biomarker visinin-like protein-1 (VILIP-1) has shown potential as a marker of MCI progression to AD [3]. VILIP-1 is abundantly expressed in the brain and is excessively released because of neuronal degradation [13]. Although increased CSF levels of VILIP-1 are not specific to AD, they have shown to correlate well with AD progression and pathology [3]. The microglia derived protein YKL-40's role in AD is not fully elucidated. It is speculated that YKL-40 increases with neuroinflammation [14]. However, apart from AD, increased levels have been found in CSF after stroke, other neurological disorders, and normal aging [14], [15], [16], [17]. This indicates that YKL-40 is more a general marker of neuroinflammation/astroglial activation secondary to many different etiologies, including Aβ pathology [18].

We hypothesized that the concentrations of markers of neuronal and synaptic damage and astroglial activation would decrease in CSF in patients with AD as an effect of moderate- to high-intensity EXE, because of stabilizing effects of EXE on neurons, synapses, and astrocytes, resulting in less neurodegeneration and inflammation and thereby lead to less release of the selected biomarkers in CSF.

## 2 Methods

### 2.1 Study population

Two-hundred community-dwelling patients with clinically diagnosed mild AD according to National Institute of Neurological and Communicative Disorders and Stroke and the Alzheimer's Disease and Related Disorders Association (NINCDS-ADRDA) criteria [19] and a Mini-Mental State Examination >19 were included and randomized to either a control group with treatment as usual or a 16-week 60-minute three times per week moderate- to high-intensity aerobic physical exercise (treadmill, stationary bike, and cross-trainer) group, in groups of four to six subjects per group with an educated trainer. For detailed description of the intervention used and study participant enrollment, see Hoffmann et al. [7], [20]. All subjects donated a blood sample before and after intervention and were tested at baseline and at 16 week follow-up with a comprehensive battery of tests of CF, activities of daily function, quality of life, physical activity, and neuropsychiatric symptoms. For details of inclusion and exclusion criteria, methods for inclusion, and cognitive and physical tests, see Steen Jensen et al. [9]. A subgroup of 56 subjects recruited from three of eight centers had a lumbar puncture performed to collect CSF samples before and after intervention. Samples where centrifuged on acquisition at 2000 *g* and stored in 250 μL aliquots at −80°C until use. The baseline characteristics of the study population can be seen in Table 1.

**Table 1:** Baseline characteristics of the study cohort

	Controls (*n* = 26)	Intervention (*n* = 25)	*P*-value∗	High exercise (*n* = 16)	*P*-value†
Age, y‡	68.9 (8.05)	68.2 (6.94)	.211§	68.3 (7.71)	.60§
Gender, *n* (%)			.15‖		.13‖
Male	19 (73)	13 (52)		8 (50)	
Female	7 (27)	12 (48)		8 (50)	
Characteristics					
Disease duration, y‡	1.7 (1.09)	1.1 (0.97)	.11§	1.3 (1.00)	.23§
MMSE‡	25.4 (3.66)	25.4 (2.96)	.29§	24.9 (2.80)	.16§
Education level, y‡	13.0 (2.96)	13.4 (2.81)	.622§	13.4 (2.73)	.665§
BMI	23.3 (3.56)	25.3 (4.42)	.24§	25.1 (4.37)	.21§
Medication					
Alzheimer's medication users	23	25	.08‖	16	.216‖
Antidepressive users	6	5	.789‖	3	.945‖
Antipsychotic users	1	0	.322‖	0	.612‖
Baseline outcome measure‡					
NFL (pg/mL)	1494.9 (634.85)	1392.1 (467.19)	.42§	1487.3 (469.89)	.45§
Ng (pg/mL)	524.4 (292.66)	628.2 (338.68)	.42§	660.3 (371.27)	.25§
YKL-40 (ng/mL)	168.4 (66.7)	181.0 (11.8)	.28§	187.7 (17.2)	.16§
VILIP-1 (pg/mL)	98.5 (70.3)	113.1 (74.1)	.95§	111.1 (73.9)	.83§

Abbreviations: BMI, body mass index; MMSE, Mini-Mental State Examination; NFL, neurofilament light; Ng, neurogranin; YKL-40, chitinase-3–like protein 1; VILIP-1, visinin-like protein-1.

∗ Controls versus intervention.

† Controls versus high exercise.

‡ Given as mean ± standard deviation.

§ Independent *t*-test.

‖ Chi-squared test.

### 2.2 Samples

CSF samples from 51 patients of the 56 possible CSF samples, subjected to 16 weeks of moderate- to high-intensity EXE (exercise group) or treatment as usual (control group), were analyzed for the concentration of Ng (UniProt #: Q92686), VILIP-1 (UniProt #: P62760), NFL (UniProt #: P07196), and YKL-40 (UniProt #: P36222), at baseline and 16 weeks follow-up.

### 2.3 Assays

CSF Ng concentration was measured using a previously published in-house Meso Scale Discovery assay as published in Kvastberg et al. [21] and De Vos et al. [22]. Commercially available kits were used for measuring the concentrations of VILIP-1 (Human VILIP-1 ELISA; BioVendor GmbH, Heidelberg, Germany), NFL (NF-light ELISA; IBL international, Hamburg, Germany), and YKL-40 (Quantikine ELISA Human Chitinase-3–like 1; R&D systems, MN, USA) by following the manufactures enclosed procedure, in technical duplicates. All measurements per assay were performed in one round of experiments using one batch of reagents for each biomarker by board-certified laboratory technicians who were blinded to clinical data.

### 2.4 Statistics

The baseline characteristics were compared between the two groups using *t*-tests. The baseline versus follow-up concentrations were analyzed using paired *t*-test within each group, respectively, and the mean change from baseline in CSF biomarker concentrations was analyzed by Student *t*-test. These analyses were done both as an intention-to-treat analysis where all participants were analyzed and as a per-protocol analysis where only the subjects that exercised with a mean intensity of >70% of maximal heart rate (HR) and attended > 80% of the sessions (high-exercise subjects) were included. The significance level was set to .05. No post hoc statistics were used.

## 3 Results

**Fig. 1:**
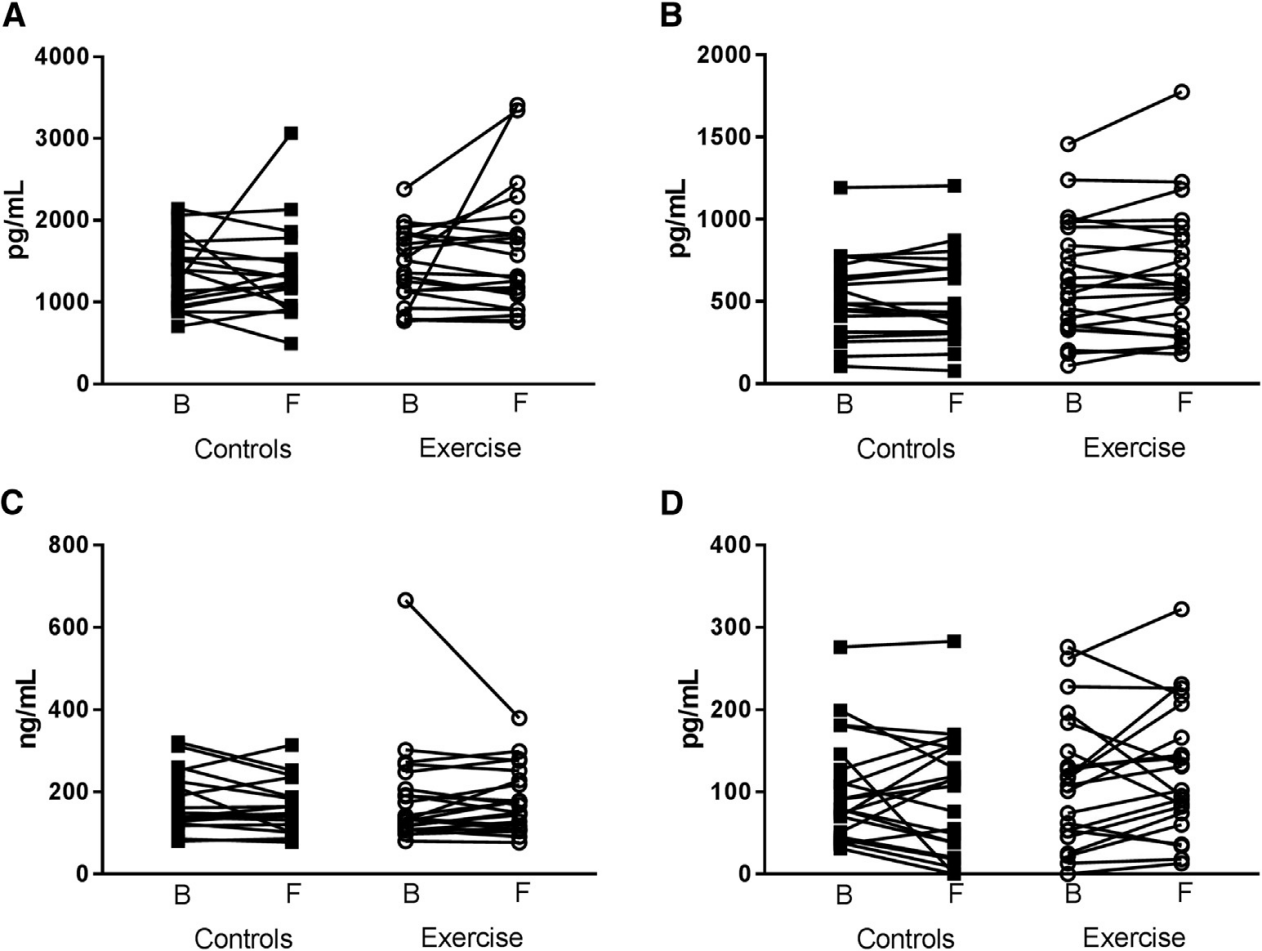
Spaghetti plots of concentration of biomarkers in cerebrospinal fluid at baseline and at 16 weeks follow-up in outcome measures. (A) Neurofilament light (NFL), (B) neurogranin (Ng), (C) chitinase-3–like protein 1 (YKL-40), and (D) visinin-like protein-1 (VILIP-1). B: Baseline and F: 16-week follow-up.

**Fig. 2:**
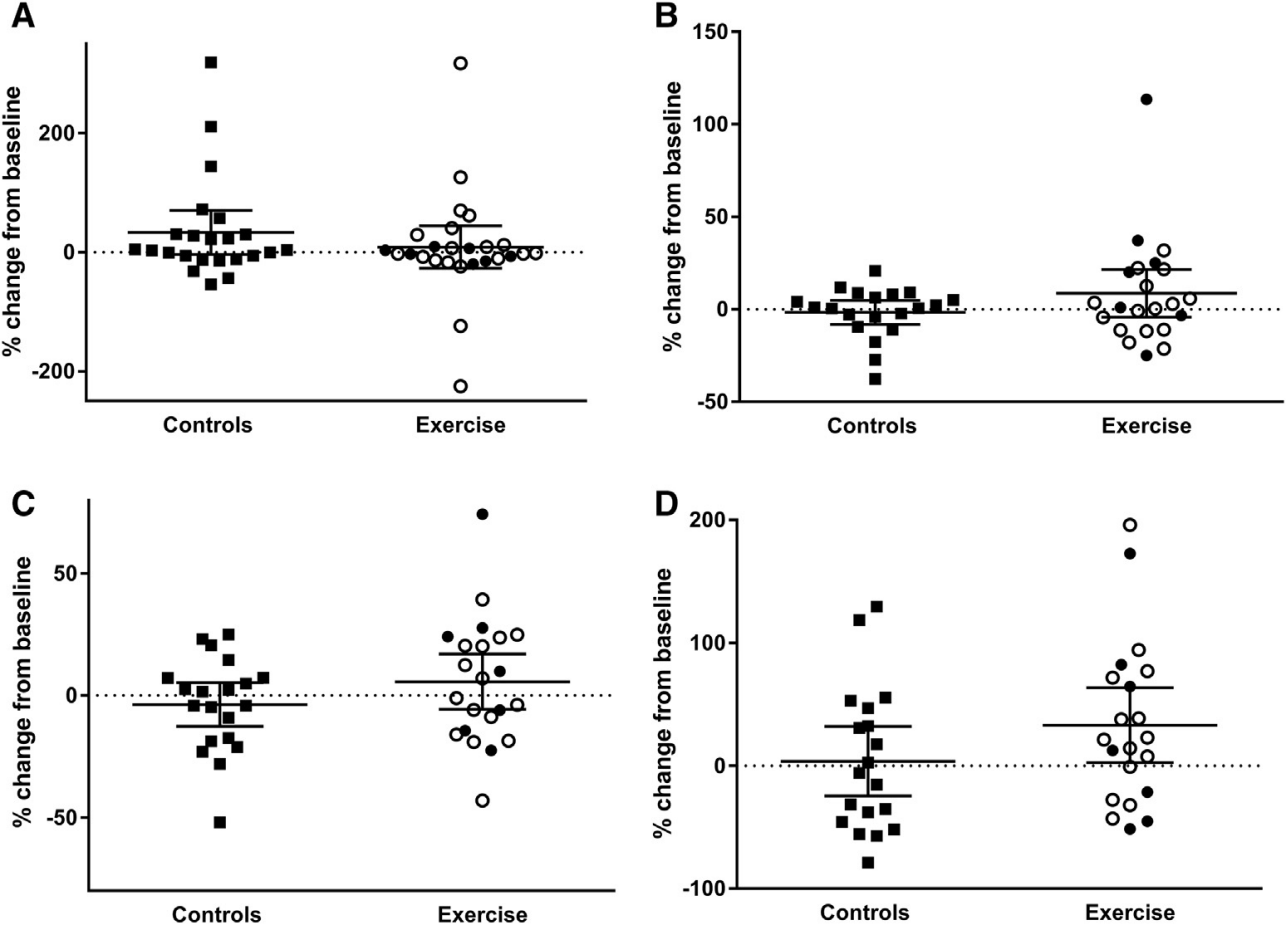
Scatterplot of relative changes from baseline to 16 weeks follow-up in outcome measure in percent. (A) Neurofilament (NFL), (B) neurogranin (Ng), (C) chitinase-3–like protein 1, and (D) visinin-like protein-1 (VILIP-1). Closed squares: controls, open circles: exercise group, and closed circles: high exercise. Displayed as mean with 95% confidential interval.

There were no significant differences in baseline characteristics between control and intervention groups (both all and high-exercise subjects) and in concentrations of VILIP-1, NFL, Ng, and YKL-40 in CSF between the intervention group and control group (Table 1). Furthermore, there was no significant difference at baseline between control and the high-exercise group with regard to the concentrations of VILIP-1, NFL, Ng, and YKL-40, as shown in Table 1. There was no statistical significant difference in the concentration of the biomarkers in CSF between baseline and 16 week follow-up in the two groups, respectively, see Fig. 1, or in means change from baseline comparing the two groups. Fig. 2 displays the relative difference between the groups in percent ([follow-up minus baseline]/baseline) × 100 in the biomarkers measured. In three out of four biomarkers analyzed, there was a trend in relative change from baseline toward an increased level in CSF after 16 weeks of intervention in the exercise group. However, the variation in the changes in both groups was large. From the controls, group coefficient of variation (CV) was calculated for each assay, to check assay stability. The assay CV ranged from 3% to >30% (NFL: 17.9, Ng: 8.6, YKL-40: 2.9, and VILIP-1: 32.3).

## 4 Discussion

In this study, we investigated the effect of an intervention of 16 weeks of moderate- to high-intensity EXE in patients with AD on selected biomarkers of neuronal and synaptic integrity and neuroinflammation. The biomarkers investigated in this study are all found in CSF under normal physiological conditions, and the levels found correspond well with findings in previous studies [10], [14], [16], [23].

Increased CSF levels of the biomarkers NFL, Ng, YKL-40, and VILIP-1 have been found in AD and correlate with neurodegeneration, suggesting a potential use as a surrogate marker of synaptic and neuronal loss [3], [22], [23], [24]. Exercise and physical activity have been linked to cognitive preservation and structural and functional neuronal changes in humans [25]. However, the underlying neurobiology is unknown. To gain new insight into the molecular pathology in AD and possible changes with physical exercise, several pathophysiological pathways can be explored. The markers selected in this study are recently investigated biomarkers of AD, and very little is known of their ability to reflect changes in the rate of neurodegeneration.

We hypothesized that EXE would have a stabilizing effect on the neurons and a dampening effect of astroglial activation resulting in less release to the CSF of the biomarkers investigated. However, we found no significant effect of EXE on our selected biomarkers. In a previous study, we also did not find effect on Aβ species and tau/p-tau [9]. These markers, however, may provide novel insight into AD pathology and its progression [16], [18], [26]. The biomarkers reflecting specific AD pathology in CSF (Aβ and tau/p-tau) change over the disease course in a pattern that does not reflect neurodegeneration in a simple way making them less suitable for longitudinal markers of disease progression [27], [28]. Because synaptic integrity plays a central role in CF and synaptic loss has been shown to correlate well with cognitive decline [29], biomarkers of synaptic function may be of value in monitoring neurodegeneration, not only in AD but also in other neurodegenerative diseases [29].

As mentioned earlier, the studied biomarkers could be target biomarkers for measuring neurodegeneration in patients with AD, including effects of drug candidates and interventions on AD pathophysiology. However, no changes were found in biomarker levels in our 16-week aerobic exercise intervention. The most straightforward interpretation of these results is that they do not support any modulatory effect of physical exercise on AD pathophysiology, as measured by selected biomarkers of neuronal and synaptic integrity. The lack of a modulating effect of exercise on our selected biomarkers might have other explanations. Animal studies in mice suggest that environmental stimulation, including access to running wheels, improved cognition and resulted in significant higher levels of brain Ng [30]. This discrepancy may be because of full-length Ng is measured in the brain, whereas in the CSF, there are several endogenous cleavage products of Ng measured [21]. Also YKL-40 has previously been studied in relation to exercise. In a human study, the postexercise serum concentration of YKL-40 was found to be upregulated both in RNA expression and in circulating protein concentration [31]. However, in that setting, the elevated YKL-40 levels may have been because of muscle contraction. One animal study found that synapse-plasticity proteins, including NFL, were upregulated in exercising rats [32], and previous studies have shown that exercise promotes neurogenesis in the adult mouse brain [33], [34]. Taken together these animal studies suggest that exercise has a beneficial effect on synaptic and neuronal integrity thereby promoting brain plasticity. The reason for the negative results in our study could be because of insufficient duration of the exercise. It could be argued that 16 weeks of EXE is not a long enough intervention to permanently alter the synaptic function in the brain. Furthermore, any exercise mediated effect on the synaptic level in AD could be so transient that the effect is not able to manifest itself permanently or that the effect wears of as soon as the subjects stop exercising. In addition, information on changes of these biomarkers with interventions or longitudinal changes is scarce. At the stage of the disease in which the subjects included in this study were studied, we do not know if it is reasonable to expect changes in biomarkers reflecting neurodegeneration. The neurodegeneration may be too advanced for a signal to be picked up, if only subpopulations of neurons respond to EXE.

A further limitation of our study is the small number of subjects. We had CSF available from 51 patients, of which 26 were randomized to the control group. Of the 25 exercise participants, 16 met the criteria “high-exercise subjects.” Restricting the analysis to high-exercise subjects compared with controls, did, however, not change the overall negative findings. However, a power calculation (80% power) showed that we had sufficient subjects in each group for detecting a 10% change in CSF concentration, based on the previous studies, for Ng (eight subjects needed), YKL-40 (four subjects needed), and NFL (nine subjects needed). Only our VILIP-1 measurement is underpowered because 37 subjects were needed to display a 10% changes in CSF concentration. From Fig. 1 and the interassay CV, we can conclude that the assays are fairly stable, and results are reproducible.

The use of EXE as a therapeutic approach for AD and other dementias is an attractive add-on to conventional medical treatment, and there seems to be a positive effect on cognition [35]. The underlying effect is, however, largely unknown [36], and more attention must be paid to the molecular mechanism to understand and optimize this type of treatment. Therefore, more studies investigating the effect of type and length of physical exercise are needed to elucidate the molecular effect of EXE in AD.

**Research in context**

1. Systematic review: Based on our previous finding in other areas of the effects of physical exercise biomarkers, we know that there is a beneficial effect of physical exercise on memory, neuropsychiatric symptoms, and physical function. However, we have not found the underlying molecular mechanism.
2. Interpretation: We interpret our results as a clue to finding the molecular mechanism, in understanding effect of exercise in patients with Alzheimer's disease (AD). Although our results are not a positive find, we now know more exercise as a stabilizing agent on synapse stability and integrity.
3. Future directions: The overall conclusion is that exercise does have a beneficial effect on the selected biomarkers; however, we recommend further studies in other areas affected by AD that could be implicated in the effect of physical exercise.
